# Artworks as dichotomous objects: implications for the scientific study of aesthetic experience

**DOI:** 10.3389/fnhum.2015.00295

**Published:** 2015-06-09

**Authors:** Robert Pepperell

**Affiliations:** Cardiff School of Art & Design, Cardiff Metropolitan UniversityCardiff, UK

**Keywords:** art, aesthetics, picture perception, neuroaesthetics, dichotomy, ambiguity

## Abstract

This paper addresses an issue that has been studied from both scientific and art theoretical perspectives, namely the dichotomous nature of representational artworks. Representational artworks are dichotomous in that they present us with two distinct aspects at once. In one aspect we are aware of what is represented while in the other we are aware of the material from which the representation is composed. The dichotomy arises due the incompatibility, indeed contradiction, between these aspects of awareness, both of which must be present if we are to fully appreciate the artwork. Examples from art history are given to show how artists have exploited this dichotomy in a way that conditions our response to their work. I hypothesize that the degree of manifest dichotomy in a work determines the strength of its aesthetic effect, and propose this could be experimentally tested. I conclude that scientific studies of aesthetic experience should take the dichotomous nature of artworks into account.

## Introduction

**Figure 1:**
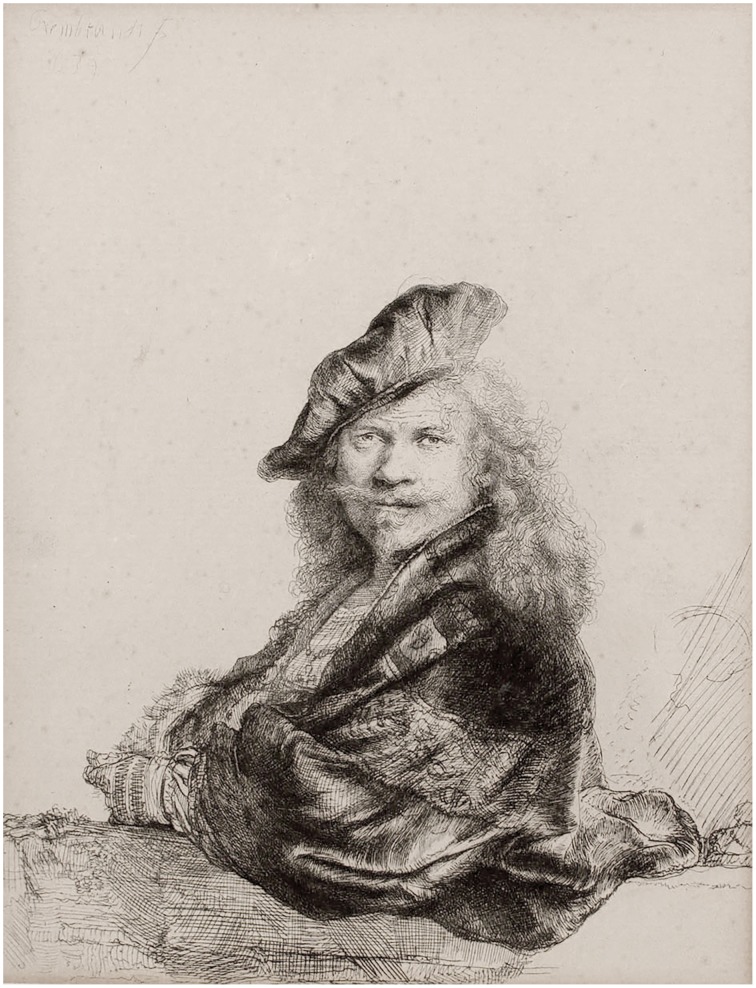
**Rembrandt van Rijn**, ***Self-portrait leaning on a stone sill***, **1639, etching on paper**. Photograph of a reproduction owned by the author.

The question of why artworks can have a strong aesthetic effect has long occupied art historians, theorists and philosophers. It has also been of great interest to psychologists and, more recently, neuroscientists. These diverse disciplines have tended to work in relative isolation however, and much still needs to be done to integrate the knowledge each has accumulated. While the task is daunting due to its scale and complexity, the potential rewards in terms of enriched understanding are great. The approach taken here is to focus on a specific feature of aesthetic experience that has been analyzed from different disciplinary perspectives and identify where the analyses agree. On the basis of such agreement new hypotheses can be generated and tested experimentally, so deepening, and broadening our knowledge in a way not possible within a single discipline alone. This theoretical article addresses one such feature, namely the dichotomous nature of visual representational artworks, as exemplified Rembrandt self-portrait seen in Figure 1.

Most of us looking at this work will be aware that it has two discrete aspects. We see a man, the artist himself, posed rather confidently in manner that alludes to portraits by Renaissance artists like Raphael and Titian. But we are also aware this is not a man but an etching composed of countless lines printed onto paper. This is the dichotomy. It does not feel as if we experience these discrete aspects alternately or exclusively, i.e., a man in historical costume and then lines of ink on paper, or vice versa.^1^ In purely logical terms this is odd. A single object appears as two quite distinct things.

Turner's *Rain, Steam, Speed* (National Gallery, London, 1844, Figure 2) presents us with a mass of vigorously applied paint, the handling of which pronounces its material properties. We also see a locomotive engine pulling carriages across the Maidenhead Railway Bridge through heavy rain. The paint here functions both as matter spread over a surface and as sky, brick, steam, metal, water, clouds, and fields. The dichotomy is more evident when the work is seen in person because the materiality of the surface “interferes” with our recognition of the forms. This can be witnessed to some extent in the detail of the painting shown in Figure 3. Seen at closer quarters the engine hovers between appearing as solid metal and buttery paint and the poor passengers in the open-top carriages almost dissolve into gray blobs. If we focus too closely on a single patch of paint the object it forms disappears and with it the dichotomous effect.

**Figure 2:**
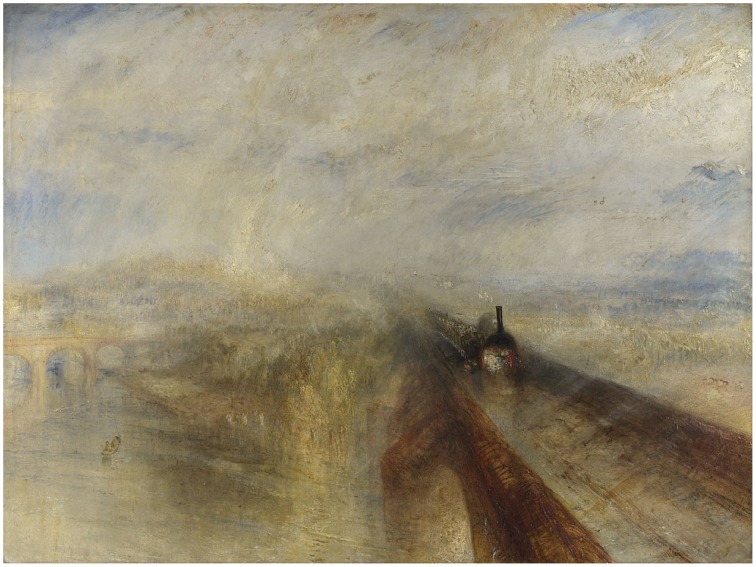
**Joseph Mallord William Turner**, ***Rain, Steam, Speed: The Great Western Railway***, **1844, Oil on Canvas, National Gallery, London**. Turner Bequest, 1856. © The National Gallery, London.

**Figure 3:**
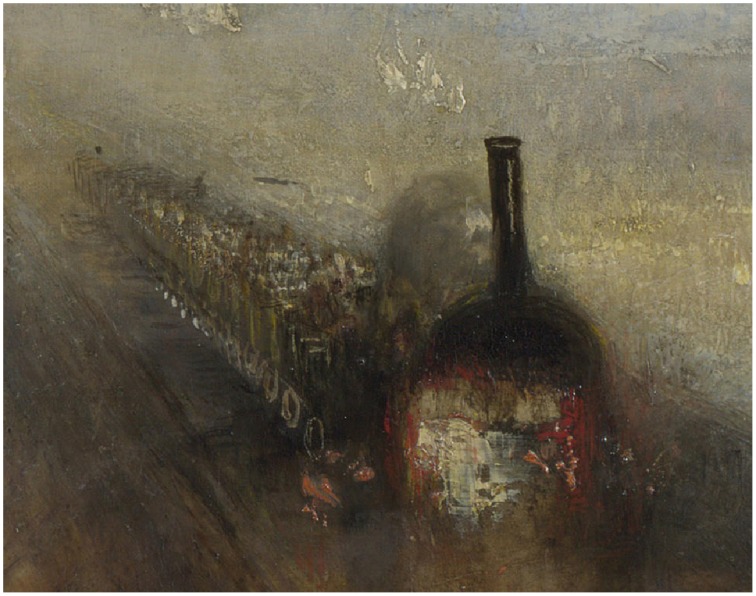
**Detail from Joseph Mallord William Turner**, ***Rain, Steam, Speed: The Great Western Railway***. © The National Gallery, London.

**Figure 4:**
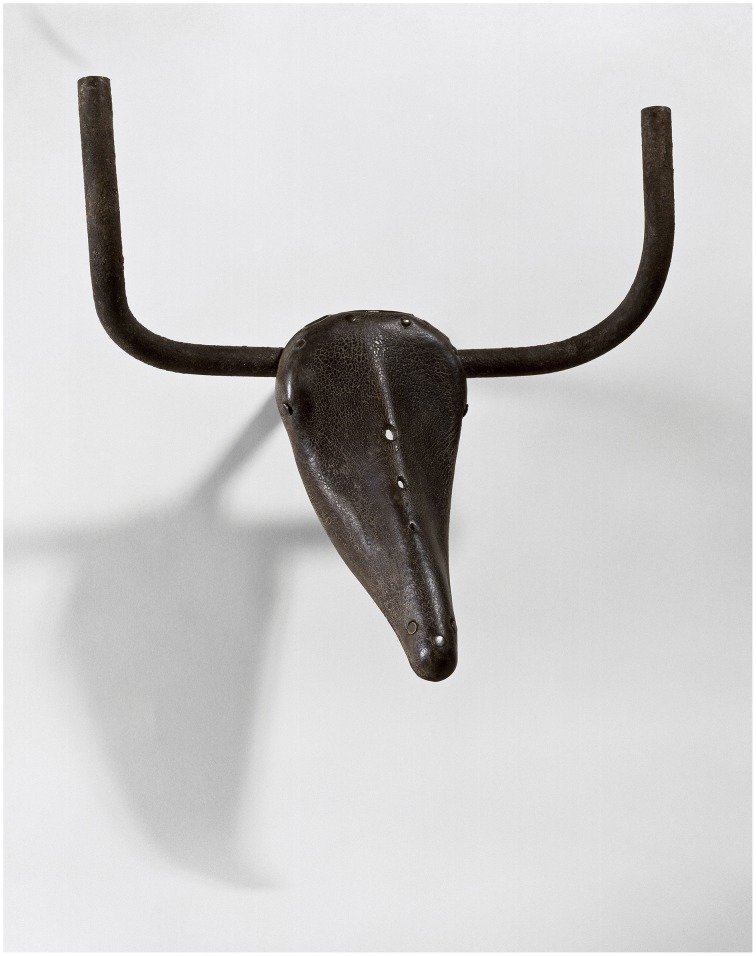
**Pablo Picasso**, ***Bull's Head*** **(Tête de Taureau), 1942, Leather and metal, 33.5 × 43.5 × 19 cm, Collection Musée Picasso, Paris**. © Succession Picasso. Photo © RMN-Grand Palais (musée Picasso de Paris)/Béatrice Hatala. DACS, London 2015.

It is not only pictures that are dichotomous in this way. When looking at the sculpture made by Pablo Picasso in 1942 titled *Bull's Head* (Figure 4) either in person or in reproduction, we perceive a bull-like form *and* bicycle parts.^2^ Picasso was clear about the importance of this dichotomy to the aesthetic impact of the piece:

Guess how I made the bull's head? One day, in a pile of objects all jumbled up together, I found an old bicycle seat right next to a rusty set of handlebars. In a flash, they joined together in my head. The idea of the *Bull's Head* came to me before I had a chance to think. All I did was weld them together& [but] if you were to see only the bull's head and not the bicycle seat and handlebars that form it, the sculpture would lose some of its impact^3^.

**Figure 5:**
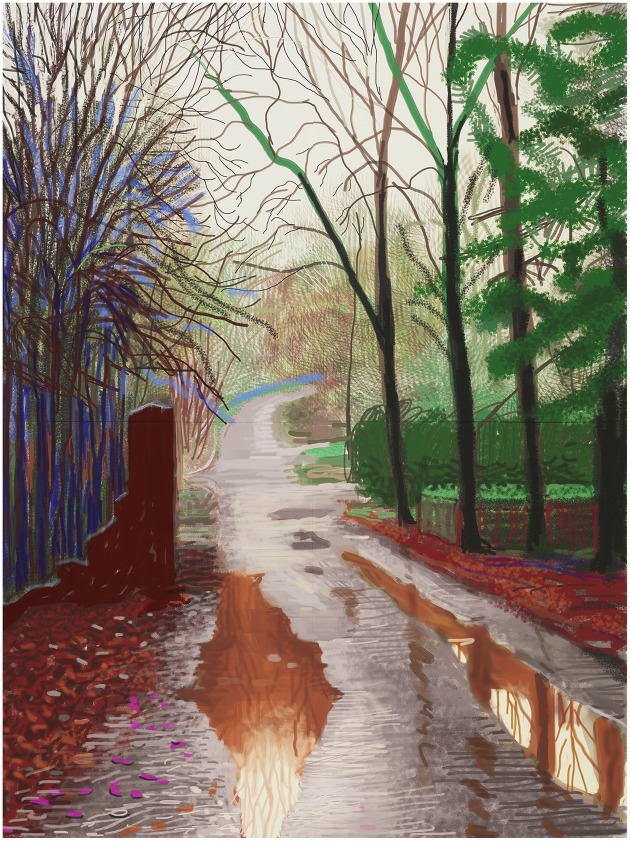
**David Hockney**, ***The Arrival of Spring in Woldgate, East Yorkshire in 2011 (twenty eleven)–29 December, No. 2***, **iPad drawing printed on four sheets of paper (46 1/2 × 35″ each), mounted on four sheets of Dibond, Edition of 10, 93 × 70″ overall**. © David Hockney. Photo Credit: Richard Schmidt.

This dichotomous effect is just as evident in a virtual medium such as computer graphics. Figure 5 shows a depiction of a British landscape by the painter David Hockney, which in its native format is composed only of illuminated pixels on an iPad. Here we see something that vividly appears as a puddle-filled country lane in spring and yet is also just as evidently a series of marks made with digital ink on a screen. The fact we see a vivid natural scene made of wiggly digital lines, just as we see Rembrandt and all the textures of his clothing composed of inky scratches, is part of what makes these images fascinating to look at, and part of what makes them works of art.^4^

The dichotomy between these two aspects of awareness is one that all representational works of art exploit because they appear both as an arrangement of materials such paint, ink, plaster, metal, stone, etc. and as whatever they represent. As we will see, many theorists have argued is a requirement of appreciating such artworks that we are aware of both distinct appearances simultaneously. They agree with Picasso that if we saw only a pair of bike parts or just the form of a bull's head much of the shock, surprise, or amusement we feel when seeing the work would be lost.^5^

It is important to note that the phenomenon being described here is not simply a kind of ambiguity or bi-stability. Ambiguous or bi-stable pictures, as generally understood in perceptual psychology, permit the viewer to alternate between two (or sometimes more) interpretations, as in the cases of the Necker cube, Rubin vase, or Duck-Rabbit, where the competing meanings are exclusive. The fact we are also aware of the patches of ink or pixels from which such pictures are composed is rarely noted. It may be possible to supress awareness of the representational aspect of a picture and see it only as a pattern of ink marks or patches of pixel color. But this is not generally how we treat such pictures; it would be the equivalent of persuading oneself to see Jastrow's duck as merely a mass of lines, and to lose the object altogether.^6^ In general, then, the dichotomous effect occurs when distinct meanings within a representational object are experienced simultaneously whereas with ambiguous or bi-stable images different meanings are experienced alternately.

“Dichotomy” is apt word to describe this state of affairs as it captures the sense of insoluble conflict between mutually exclusive but simultaneously occurring states.^7^ Works of art are not unique in this respect. Most forms of representation in which material is organized so as to evoke something other than itself will have the same character, including writing, the use of signs and symbols, photography, cinematography, computer graphics, sound recording, music, and is arguably what structures the signifier-signified relationship between sounds and meaning in language itself.^8^ But I will argue there is something special about the way artists exploit this property that is an important part of how artworks function aesthetically and which any scientific explanation of aesthetic experience will need to take into account.

The dichotomous nature of pictures and paintings has been studied in several science, arts and humanities contexts, although described using different terms. I review a number of these studies to show that despite the different disciplinary contexts there is broad agreement about how these objects function. In particular, there is a recurring suggestion that pictures and paintings induce a kind of “dual” or “split” state of mind in which we are aware of distinct and incompatible aspects of the work simultaneously.^9^ Many authors characterize this state as “impossible,” “contradictory,” or “paradoxical,” in other words as irrational. Despite the frequency with which this has phenomenon been noted, and the eminence of some of those who have noted it, there has so far been no concerted attempt to provide a scientific explanation.

Building on this previous work on the duality of pictures and paintings, I will propose there are three aspects to the dichotomous nature of representational artworks that can condition our aesthetic response: first, we are aware of the discrepancy between the matter from which the artwork is composed and what it represents; second, we are aware of discrepancies between the way things are represented in the artwork and how we would expect them to look in reality; and third, we are aware of many distinct conflicting meanings that attach to the same work at the same time. I will present some specific examples from art history that illustrate these aspects, and suggest a provisional hypothesis about how they contribute to an artwork's aesthetic impact. Finally, I will consider the implications of these observations for the scientific study of aesthetic experience.

## The dichotomous nature of picture perception

Not all artworks are pictures, and not all pictures are artworks.^10^ Yet all pictures share with all representational artworks the property of being dichotomous objects. The dichotomy has been recognized by a number of important researchers in picture perception. Pirenne (1970) talked of the “subsidiary awareness” we have of the material surface when directing our attention the contents of a picture, the importance of which he believes has been largely overlooked in theories of pictorial representation. Gibson (1978 and 1979) noted the way a picture acts both as a physical surface and a display of information about something else: “The viewer cannot help but see both, yet this is a paradox, for the two kinds of awareness are discrepant” (1979, p. 282). In similar vein, Gregory (1970) regarded pictures as occupying a contradictory “double reality” in which “they are seen both as themselves and as some other thing, entirely different from the paper or canvas of the picture.” He wrote:

Pictures are paradoxes. No object can be in two places at the same time; no object can lie both in two- and three-dimensional space. Yet pictures are both visibly flat and three-dimensional. They are a certain size, yet they are also the size of a face or a house or a ship. Pictures are impossible.^11^

In one sense, pictures are clearly not impossible because we see them all the time and have little trouble recognizing their content. But Gregory suggests our capacity to appreciate the “double reality” of pictures somehow falls outside the conventional bounds of rationality. It is not pictures in themselves that are paradoxical, contradictory, or impossible but our perceptual and cognitive responses to them.

Developmental studies tell us the capacity to appreciate this aspect of picture recognition is acquired only gradually in early life. DeLoache and colleagues investigated anecdotal reports that very young children would frequently treat objects in pictures as though they were real (DeLoache et al., 2007). Children were observed, for example, trying to step into a picture of a shoe or grasping a depicted object, indicating they were yet to appreciate the representational nature of pictures. By studying the behavior of 9-month-old children presented with vividly illustrated picture books, the experimenters were able to substantiate the anecdotal evidence and show that, in fact, such behavior is very common among children of that age. A cross-cultural comparison between middle class children raised in the USA and impoverished children from the Ivory Coast, who had far less exposure to printed pictures, showed a pattern of behavior that was remarkably similar. The researchers found that by the time the children reached infanthood, around 19 months old, the observed behavior had changed, with children tending to point to or name depicted objects in books rather than try and pick them up. They concluded that while children can recognize objects within pictures at an early age without having to specifically learn to do so it does take time and experience to realize that objects and pictures of objects are different. This capacity appears to be well-established in children by the age of around 2 years. Preissler and Bloom (2007) investigated whether children with a mean age of 30 months were able to appreciate what they called the “dual nature of pictures” and found they had the same ability to do so as a group of adults given the same tasks. However, Jolley (2008) using a different methodology argues that it is only by the age of 3 or 4 years that children fully appreciate the dual nature of pictures, which he defines as occurring when “an individual is not constrained to thinking about the picture either as a thing in itself or as a representation of another reality (the referent), but is aware of both components at the same time” (Jolley, 2008, p. 94).

Approaching the topic from the perspective of perceptual psychology, Rainer Mausfeld uses the term “conjoint representations” to denote the doubled and “mutually incompatible” meanings present at the same time within a picture (Mausfeld, p. 25). He argues both these representations are “internally locked,” that is, bound together within the cognitive system in a way that allows us to exploit the same perceptual input in two different ways. Although little understood, this process must be a fundamental feature of cognition, he suggests, because conjoint representations occur not just in picture perception but also in many other areas of human behavior. He cites several examples, including in language use, in pretend play and acting where the imaginary world inhabited by player or actor contradicts the real world in which the pretense occurs, and in art where we can impute emotional states to something we know to be a flat, static image such as a photograph or print. An etching of a crying woman by Picasso is given as an example.

Niederée and Heyer (2003) attribute the dual nature of pictures not to the objects themselves but to the psychological process by which we perceive them. In their view, the standard framework of perception cannot account for the way we experience pictures. This predicts that from a given retinal stimulus we will infer the presence of a picture as an object or what the picture represents, but not both at the same time. The fact we are aware of both aspects simultaneously may be explained, they say, by our ability to switch attention between the salient features of the representation and our residual awareness of the flat surface. Crucially, these dual aspects do not coexist in parallel but compose a single “perceptual unit” (p. 82). In other words, there is one percept containing two aspects, and it is only when both aspects are present simultaneously that picture perception proper occurs. In this way they distinguish pictures from other forms of image that lack this dual property, such as trompe l'oeil or entirely abstract paintings. Like Mausfeld, Niederée and Heyer also regard the capacity to experience duality phenomena as not being confined to pictures or art but as a more general feature of our cognitive constitution, to be found in phenomena such as the perception of mirrors and shadows. They also note that certain works of art, such as paintings by Arcimboldo (discussed below), comprise special cases of pictures that increase our awareness of their duality.

We can see that among those interested in the psychology of the perception of pictures there is recognition of their dichotomous nature, whether referred to in terms of subsidiary awareness, double reality, conjoined representations, or duality. While they do not consider artworks in detail, these studies do offer some suggestions about how the dichotomous aspects of pictures may contribute their aesthetic effects. For instance, several authors point to there being something unusual or out of the ordinary in our response to pictures that does not seem to be accounted for within the standard paradigm of perceptual theory, namely that they require us to be simultaneously aware of distinct and mutually contradictory aspects of the same object. The same observation has been made by a number of art theorists, historians, and philosophers who have focused more specifically on the dichotomous nature of paintings.

## The dichotomous nature of paintings

The dichotomous nature of representational paintings has been a longstanding topic of debate among historians, theorists and philosophers of art, and continues to be. Much discussion has centered on a claim made by Ernst Gombrich in *Art and Illusion* that it is not psychologically possible to attend to the content of a painting and its material support at the same time. Referring to the painter Maurice Denis' declaration that a painting, before being a battle horse, is first a plane surface covered in an arrangement of paint, Gombrich said:

But is it possible to “see” both the plane surface and the battle horse at the same time? If we have been right so far, the demand is for the impossible. To understand the battle horse is for a moment to disregard the plane surface. We cannot have it both ways.^12^

**Figure 6:**
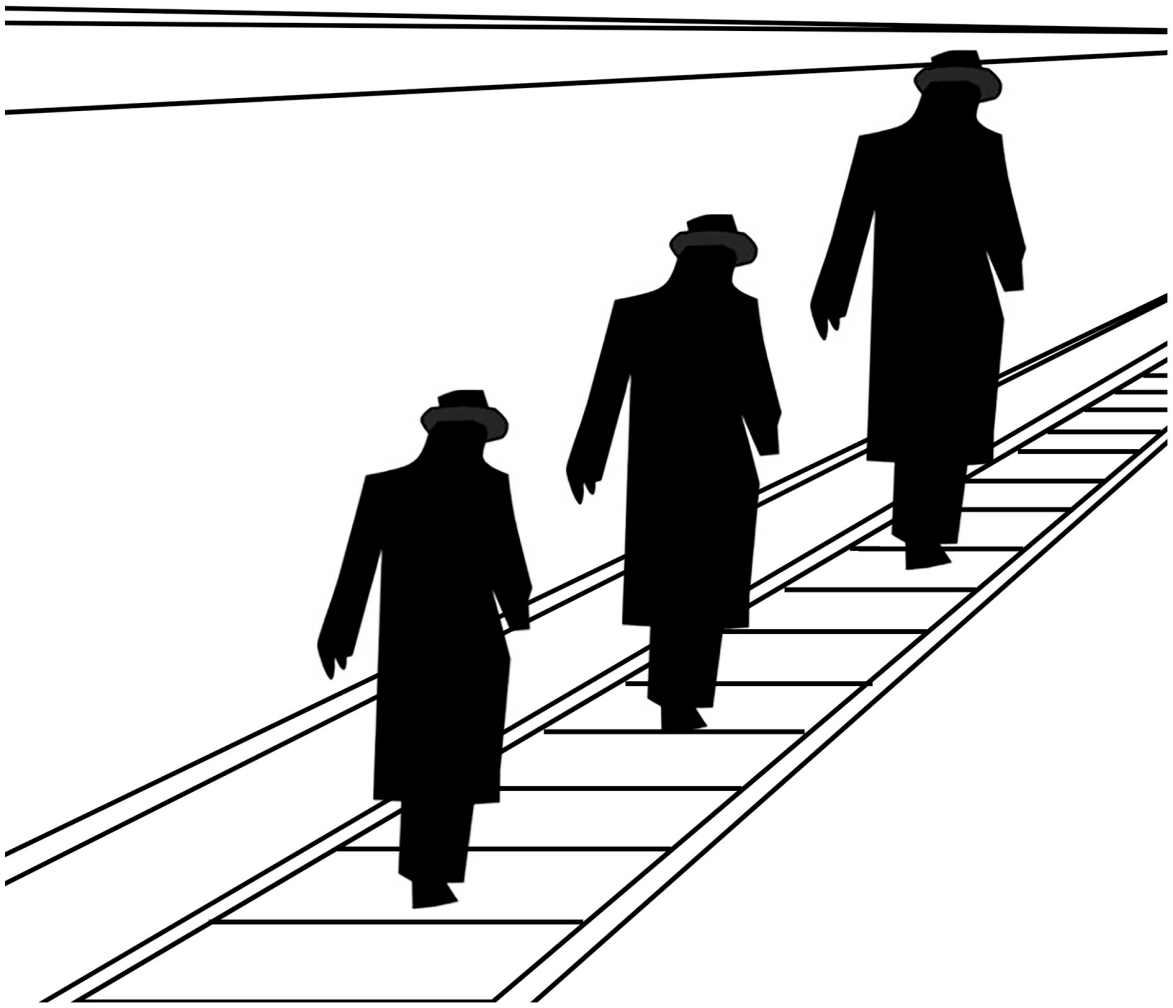
**The “men in a tunnel illusion.”** Based on the image included by Ernst Gombrich in *Art and Illusion*.

Gombrich is unequivocal. We cannot attend to both the painted surface and the battle horse at once, and the more compelling the illusion of the horse the less likely we are to attend to the substrate from which it is composed. Speaking of the “men-in-a-tunnel” illusion, a version of which is shown in Figure 6, Gombrich asserts that with some effort we might see the image as no more than a pattern of lines, and so “lose” the men and tunnel. Our perception might then revert to seeing the men and tunnel again, or oscillate between the two ways of seeing. But we cannot see the image both as a pattern of lines and as men in a tunnel at the same time, just as we cannot see the two orientations of the Necker cube simultaneously or the duck and rabbit together in that illustration.

The philosopher Richard Wollheim took exception to Gombrich's claim. He argued it was a necessary condition of seeing representations that we are simultaneously aware of the object depicted and the medium of which it is composed. He developed a “twofold” thesis in which “visual attention must be distributed between two things,” namely the surface in which the representation is inscribed and the object depicted (Wollheim, 1980, p. 213). In its original formulation, Wollheim thought of these two things as distinct experiences, but later modified this to make both parts of a single experience.

Perhaps the most durable contribution Wollheim made to the debate about pictorial perception was his deployment of the notion of “seeing-as,” derived from Wittgenstein, later elaborated into the notion of “seeing-in.” Wollheim describes seeing-as in terms of the capacity to see one thing as another. For example, when I mistake a bag on a chair for a cat I see the bag *as* a cat. In such a case, there is no twofold quality to the perception. In the case of seeing-in, however, “… I can simultaneously be visually aware of the *y* that I see in *x* and the sustaining features of this perception.” (p. 213) In support of his twofold thesis, and against what he takes to be Gombrich's position that we can only alternate between attending to the depiction and its substrate, Wollheim describes the aesthetic experience of looking at great paintings:

… in Titian, in Vermeer, in Manet we are led to marvel endlessly at the way in which line or brushstroke or expanse of color is exploited to render effects or establish analogies that can only be identified representationally, and the argument is that this virtue could not have received recognition if, in looking at pictures, we had to alternate visual attention between the material features and the object of representation.^13^

The precise way in which this dissociation between material and representation in pictures is sustained, and how the two parts of the twofold experience are related is not explained (Wollheim talks about the way artists seeks a “rapport” between them). Nevertheless, the twofold thesis, in various forms, is now widely accepted as a necessary condition for pictorial representation and the appreciation of representational art.^14^

Posing the question “what is a painting?” Polanyi (1970) largely follows Pirenne in distinguishing between the awareness we have of the representational content of a work and our subsidiary awareness of the canvas and brush strokes from which it is composed. He does allow that we may attend more to the representational content or the material form depending on how we view the work, whichever we choose being the subject of what he calls our “focal awareness.” Although the subject of this focal or subsidiary awareness can alternate, in order for an object to be read as a painting, in his terms, we must comprehend both. He says “a painting includes both the perspectival depth of its paint and the flatness of its canvas, these two contradictories being seen as one joint quality, and this is indeed the quality that distinguishes a normal painting” (1970, p. 229). Again, he stresses the dichotomous state this engenders: “This union is not a fusion of *complementary parts* into a whole, but a fusion of *contradictory features*. The flatness of a canvas is combined with a perspectival depth, which is the very opposite of flatness.” (p. 23, emphasis in original). What contributes to the affective power of a work is the way it can elicit deep memories, emotions or associations as we recognize the content, while at the same time we are confronted with the artificiality of the medium through which they are evoked. He argues the irreconcilable conflict that results goes beyond anything else to be found “in nature or in human affairs,” becoming what he calls “transnatural.” He concludes: “works of art are generally formed through integration of two incompatible elements, one of these being an attempted communication and the other an artistic structure that contradicts the communication.” (p. 235) It is through our experience of this incompatibility that works of art, not just visual but also theatrical and literary, have their power to move us.

In a book that, like Polanyi, asks *What is Painting?* the artist and writer Julian Bell argues that what is significant about representations is that they confront us with a contradictory sense of things that are present but also absent (Bell, 1999). He talks about the “mark” as a material object to which we assign associations, whether this is the intentional mark made by artist or the incidental mark made the skid of a tyre, or a boot print in the ground:

We see it and we see past it, or into it; it is what it is *and* a reminder of something else besides. It is when we see something in that double, ambivalent manner that we call it a mark. Seen another way, it might be so many grams of paint, or of rubber, or of a hole of such and such a depth in the ground.^15^

The philosopher Jennifer Church refers to the way we experience a kind of “double consciousness” when seeing landscape paintings:

When we see an X as Y (a painting as a landscape, say) we partake in a kind of double-consciousness, experiencing a thing in two different ways simultaneously (the painting way and the landscape way)—ways that retain their independence despite their convergence on a single object at the same time.^16^

Church's explanation of this double consciousness draws on a Kantian framework in which “… we experience different ways of seeing, or different appearances, as both conflicting and convergent whenever we are conscious of objects…” (p. 109). In her view, an object—the painting—can also have the appearance of something else—a landscape—because it is a requirement of conscious seeing in general that we conceive the different aspects from which it is possible to view a scene, but that these converge in our own single view. Our perception of the painting as an object conflicts with but also converges with its appearance as a landscape. In this way, Church retains the contradictory dualistic character of representations while seeking overall conceptual unity in the experience.^17^

In addition to what was said on this topic by psychologists, there is recognition among art theorists and philosophers of what I have termed the dichotomous nature of representational artworks. Moreover, there are clear points of agreement across these disciplines about how pictures and representational paintings function, in particular that we experience these dual aspects as bound together and distinct at the same time. It is this property that several authors have associated with contradictory or paradoxical states of mind in those who view the objects. By looking at a series of examples of works of art, as we will do in the next section, we can see how these dichotomous properties have been exploited or manipulated by artists in order to condition our responses to their work.

## The aesthetic effects of artworks as dichotomous objects

The OED offers a supplementary definition of dichotomy as something paradoxical, ambivalent, or contradictory.^18^ It is these “impossible” qualities of representations referred to by Richard Gregory that artists have been long aware of and have deliberately exploited for aesthetic effect. Guiseppe Arcimboldo's arrangements of fruit, vegetables, flowers, and other objects that metamorphose into formally posed heads can seem frivolous compared with the heavily religious subjects of his Mannerist contemporaries. But they are part of a highly cultivated tradition in European art in which the illusory and deceptive nature of painting is made the subject of painting itself.^19^

**Figure 7:**
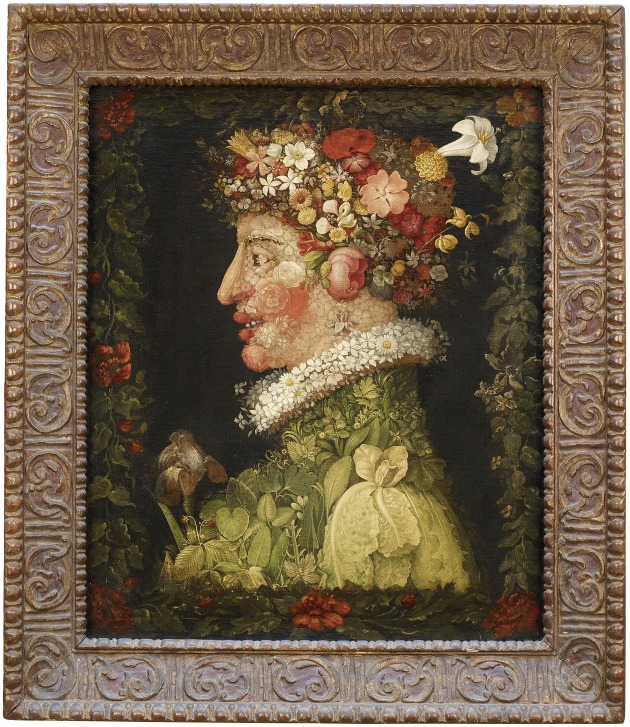
**Giuseppe Arcimboldo**, ***The Spring***, **1563, Oil on oakwood, 66 × 50 cm, Musée du Louvre, Paris**. Photo © Musée du Louvre, Dist. RMN-Grand Palais/Angèle Dequier.

Looking at the example from Musée du Louvre in Paris in Figure 7, we are aware of several distinct representational layers co-existing in the same plane: the painted surface, the cornucopia of flower heads and leaves, and the emerging figure in profile.^20^ As Niederée and Heyer note above, we immediately appreciate these distinct layers, and indeed it is necessary for the striking effect of the painting that we do, despite the inherent discrepancies between them. The cornucopia, for example, is recognizable independently from the profile, despite each being composed of the same forms and of the same paint. In one section a flower is an ear and also an arrangement of paint and in another leaves appear both as themselves and a shoulder. The fact that the profile is composed of vegetation draws attention to the mismatch between the head-like form we see before us and the way we know a real head should look. As with Picasso's bull, we perceive something that looks like a head and not like a head. The overall effect is to induce a degree of perceptual dissonance that is exciting, if not somewhat disturbing.

**Figure 8:**
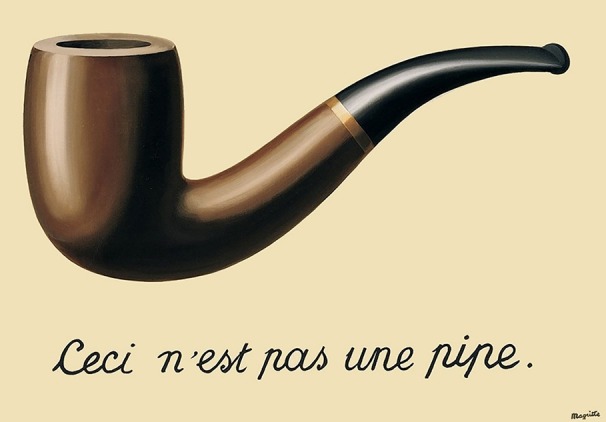
**René Magritte**, ***The Treachery of Images***, **1928/29, Oil on canvas, 62 × 81 cm, Los Angeles, County Museum**. © ADAGP, Paris and DACS, London 2015.

Perhaps the most iconic example of pictorial paradox and contradiction in twentieth century art is René Magritte's *The Treachery of Images* (Figure 8). This work manifests the slippery conceptual properties that mostly remain submerged in our daily dealings with pictures, but which Magritte provocatively exposes through his title. *The Treachery of Images*, better known as “This is not a pipe,” is at once a bald statement of the obvious and a crafty self-denial, a patently true proposition that also undermines its own premise, a visual manifestation of the paradox wherein the statement “This statement is false” is both true and false, and a darkly humorous thesis on the indeterminacy of language and meaning. It is the “unreal” manner of its handling, as blandly illustrative, which alerts us to the dissonance between what we would expect to see in the presence of a real pipe and the pipe-like shape we actually confront. The philosopher Michel Foucault did his best to unravel the mystery of the painting by presenting it (not entirely convincingly, in my view) as a calligram—a word-image of the kind associated with Guillaume Apollinaire, the poet, critic and early supporter of the Cubist movement (Foucault, 1983). But like all true paradoxes, *The Treason of Images* cheerfully resists any attempt at rationalization and stubbornly asserts the fact that the shape above the words is clearly a pipe, and yet—being confected from paint—is also not a pipe.

**Figure 9:**
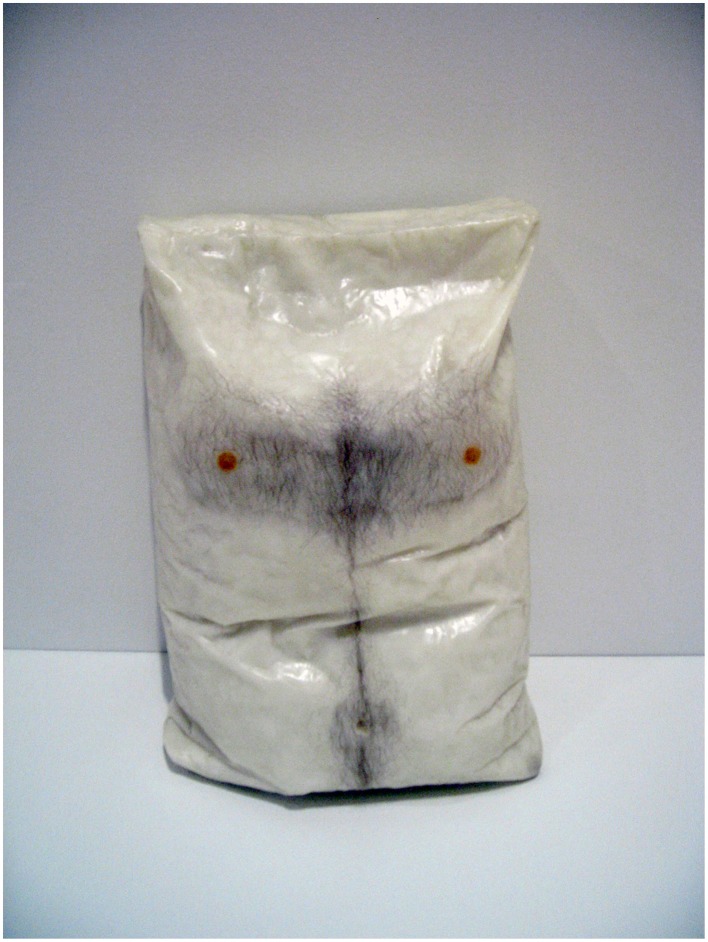
**Robert Gober**, ***Untitled***, **1990, Beeswax, pigment, and human hair, 60 × 44 × 29 cm, San Francisco Museum of Modern Art**. Photograph by the author. © Robert Gober, Courtesy Matthew Marks Gallery.

Robert Gober's *Untitled*, from San Francisco Museum of Modern Art, consists in a plastic sack molded in beeswax and adorned with paint and human hair to look like a nude male torso (Figure 9). The work has an immediate visceral impact when seen in the “flesh” as the perceptual system struggles to process an object that is a sack and torso at the same time, objects that in normal circumstances are quite distinct. We are put in mind of the “body bag,” a conveyance for mutilated corpses, of which this appears to be an example. Yet at the same time it is too witty a statement to be macabre. The creases take on a double function, as folded flesh and plastic; the hair is real and yet at the same time stands in for hair; the waxiness of the material's surface evokes greasy skin but is determinedly wax; the dots are paint and flushed nipples. Seeing an object that is vividly a body but just as vividly not a body is the perceptual equivalent of being given something and having it taken away at the same time. Coping with this dissonance must be a challenge for the cognitive system, yet is a crucial part of the overall aesthetic effect of the work.

**Figure 10:**
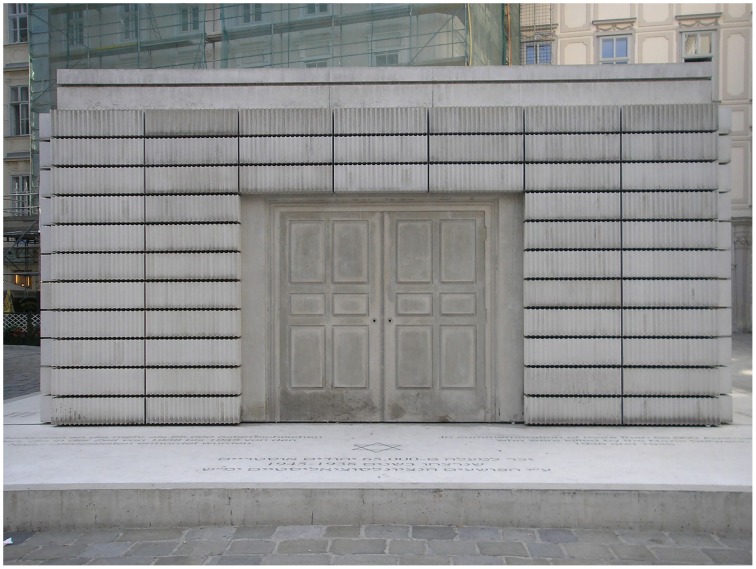
**Rachel Whiteread**, ***Judenplatz Holocaust Memorial***, **2000, Concrete, Judenplatz, Vienna**. Image source: Wikimedia Creative Commons. http://commons.wikimedia.org/wiki/File:Holocaust_Mahnmal_Vienna_Sept_2006_001.jpg. By Gryffindor.

In a series of works made since the late 1980s, the sculptor Rachel Whiteread produced casts of the inverse or negative space around objects. In this way she famously materialized the interior of an entire Victorian house in London in 1993 and was commissioned in 1996 to produce a memorial to the Austrian Jews who died in the Holocaust of World War II (Figure 10). The work was eventually unveiled in 2000 and stands in Judenplatz in the city center. Also known as the “Nameless Library,” the memorial appears at a distance to be an ominous bunker robustly constructed from blocks of masonry, but when approached turns out to be a finely cast impression of hundreds of books on library shelves. These books, and the imprint of heavy doors, all occupy the space we would occupy if standing inside this imaginary library. Everything is present only by negation. Concrete, which is the epitomic material of permanence and solidity, conjures up a sense of loss and invisibility. The fineness of the casting, with the detail it picks up, only serves to reinforce the disparity between what we imagine to be present and what we really see, between a real door and an antithesis of a door.

The dichotomies manifested these artworks are of three distinct but related kinds. First, in each work we are aware of the disparity between its material constitution and the objects it represents. This is clearly demonstrated in the Rembrandt etching, where we see both the ink marked paper and the human figure, or in the Hockney where the puddles and trees are also wiggly lines of digital ink. In the Gober piece, the wax is formed so as to represent a body and a bag, much as the metal and leather in Picasso's bull's head is formed into bicycle parts and animal parts. In Whiteread's monument the concrete stands as itself and also as book-filled shelves and doors, albeit rendered in reverse. Arcimboldo plays an even trickier game by presenting us with paint that is also flowers and, at the same time, with paint that is both flowers and a human figure.

The second kind of dichotomy manifest in these works arises from the disparity between how we expect an object to look and how it actually looks in the work. We see this in the Gober, where the hair and nipples evoke a human form while the bag-like shape confounds our expectation of how a human form should appear. Magritte deliberately rendered his pipe in a mannered and banal way. We know immediately that real pipes do not look like this, yet this does nothing to lessen our capacity to recognize it as a pipe.^21^ The profile in the Arcimboldo is face-like enough, but also sufficiently different to remind us it is not a face. The putative bull's head in the Picasso looks quite different from a real bull.^22^ The effect of this kind of dichotomy is to strongly evoke the presence of an object and at the same time remind us of its absence.

The third way in which artworks manifest their dichotomous nature lies in their capacity to elicit a multitude of distinct and contradictory meanings in the mind of the viewer. It is characteristic of great works of art that they cannot be narrowly or precisely defined. Some researchers have argued certain works of art are great precisely because they evoke multiple or incomplete meanings (Zeki, 2004). There is still no agreement among scholars about the correct interpretation of Diego Velazquez's epic studio set piece, *Las Meninas* (1656). But partly because it encourages so many interpretations, the painting is often regarded as the finest in the European tradition. It is this capacity for conveying multiple meanings that Picasso referred to in a remark to his biographer Roland Penrose: “when looking at a picture, one should say that the more associations it can open up the better” (in Cowling and Roland, 2006, p. 264). Whether these diverse meanings occur in the mind of the viewer at precisely the same time, gradually accumulate through contemplation, or fade in and out of awareness in turn is hard to determine from introspection alone. Nonetheless, the fact that each work can support multiple dichotomous meanings is an important part of their overall aesthetic effect.

**Figure 11:**
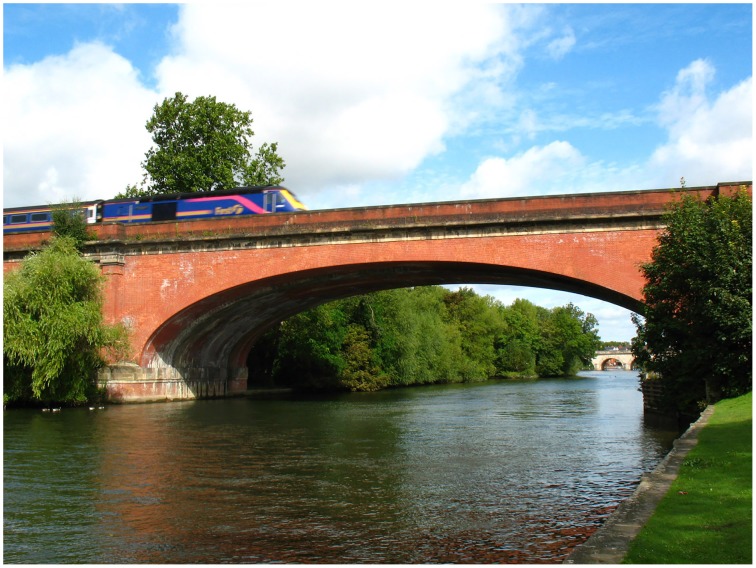
**Photograph of a train crossing Maidenhead Bridge**. By kind permission of Stephen Dawson. © Stephen Dawson, 2007.

The way artworks exploit these three kinds of dichotomy may be one of the factors distinguishing them from representational objects in general. For while all pictures and representational objects engender multiple and contradictory states of perception by their dichotomous nature, works of art do this to a greater extent. The photograph in Figure 10 of the Holocaust Memorial is, like most photographs, a dichotomous object. Yet it is unremarkable in itself when compared to the experience of seeing the memorial in person. Or consider Figure 11 showing a modern train crossing the same railway bridge depicted by Turner in Figure 2. Although pleasant enough, it has none of the difficulty, expressiveness, or atmosphere elicited by Turner's painting of the same subject. We have little reason to pay attention to the fabric from which it is composed, nor does it surprise us as a depiction of how a train would look crossing a bridge.

**Figure 12:**
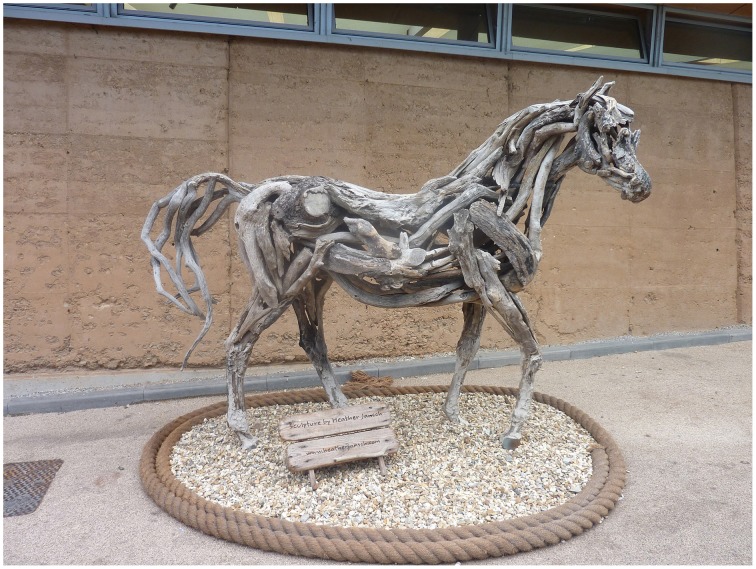
**Photograph of a sculpture of a horse made from driftwood by Heather Jansch**. Photograph by the author, 2010.

Figure 12 shows a horse sculpture made by the artist Heather Jansch, which is installed in the grounds of the Eden Project in Cornwall in the UK. It has similarities with Picasso's bull in being an animal form composed of discarded objects. And as with the Picasso, the effect of seeing the horse in person is striking because we are aware of the co-presence of the horse form and the driftwood. What makes it a less aesthetically compelling work of art, I would argue, is its relative lack of dichotomous properties, of conflicting or surprising associations. The form of the horse matches quite closely what we would expect of a horse, unlike in the Picasso where the bull form deviates to a greater extent from our expectation of how a bull should look.

For many people, of course, the Jansch sculpture will be aesthetically preferable to the Picasso precisely because the arrangement of matter follows more closely the expected form of a horse. In this sense it is more “realistic” or recognizable, and appears to show greater evidence of skill in its construction. It is probable that the level of expertise of the viewer will be an important factor in this judgment, with art experts being inclined to favor the Picasso because it places greater demands on imaginative resources and because it has deeper poetic resonance (the leather of the seat evokes the skin of the animal, we think of holding the handle bars and “taking the bull by the horns,” of Picasso conjuring up a potent symbol of Spanish vitality from the among the privations of wartime Paris, etc.). For all its skilful construction, the wooden horse fails to ignite as many diverse associations, and therefore ranks as a lesser work of art.

## The dichotomous nature of artworks and aesthetics

According to the literature reviewed here from both scientific and art theoretical perspectives there is general agreement about the dichotomous nature of pictures and paintings, albeit referred to using different terms. As viewers we are aware of both their material and representational properties simultaneously, despite the fact these properties are distinct and mutually incompatible. I have made a more specific claim about works of art in general, which is that they manifest this dichotomy to a greater extent than non-works of art and that they do so in three ways: by drawing attention to the disparity between their material constitution and their representational content, by alerting us to a further disparity between the way objects are represented and how we expect them to appear, and by encouraging awareness of multiple diverse and conflicting associations. All these properties are evident in the art historical examples presented here. Based on these observations I offer the following hypothesis: that our aesthetic experience of artworks is determined, in part, by our awareness of their dichotomous properties. This hypothesis predicts a correlation between manifest degree of dichotomy and aesthetic effect, such that objects manifesting greater degrees of perceived dichotomy will elicit a correspondingly stronger aesthetic experience.^23^ The hypothesis further predicts higher levels of art expertise will be a factor in preference for greater degrees of perceived dichotomy.

Implicit support for these proposals can be found in the literature linking ambiguity to aesthetic appreciation. Ambiguity, as discussed in much of the literature on the psychology of aesthetics, refers to vagueness or the capacity of the same material to carry multiple meanings. It has long been regarded as an important mechanism for heightening the aesthetic impact of works of art (Empson, 1930; Kaplan and Kris, 1948; Berlyne, 1971). In ambiguous works these meanings are open to varying interpretations and may change or fluctuate over time, or be resolved in a way that dispels the ambiguity. As noted at the outset, the dichotomous state is characterized by the simultaneous presence of conflicting meanings; these are not open to reinterpretation or resolution if the state is to remain. Some applications of the term ambiguity, however, do seem to have an affinity with the dichotomous properties of artworks discussed here. In the context of literary criticism, for example, William Empson (1930) described seven kinds of ambiguity used by poets and writers for literary effect, among them the use of “full contradiction, marking a division in the author's mind.” Empson's ambiguous types were further elaborated by Kaplan and Kris (1948). They identified, among others, “disjunctive ambiguities” containing several alternative and mutually exclusive meanings, and “integrative ambiguities” where manifold meanings interact to form a complex and shifting pattern of overall sense. These different kinds of ambiguity are employed in literary works to enhance their aesthetic value.

Berlyne (1971) discussed the way cubist paintings can present the viewer with contradictory cues, where one segment of the painting can belong at the same time to two objects. These cues can be registered simultaneously, in which case they generate incongruity or conflict, or they can suddenly alternate in meaning, with one interpretation replacing another, giving rise to an increase in arousal or surprise. More recently, Jakesch and Leder (2009) showed that moderate levels of perceived ambiguity or dissonance in modernist works of art are preferred to those with low levels. Excessive levels of dissonance, however, reduced this positive effect. A further study by Jakesch et al. (2013) not only found that ambiguous artworks were rated more positively than non-ambiguous ones but the fact they were perceived as being harder to interpret made a contribution to this positive effect. This suggests the perceptual problems posed by ambiguous or dissonant artworks are experienced as beneficial to their aesthetic value. Muth et al. (in press) reported a robust correlation between degrees of ambiguity in artworks and aesthetic preference, with greater perceived ambiguity leading to higher ratings for liking and for interest. This positive aesthetic rating was also enhanced if the viewers felt able to resolve perceptual problems and gain insights into the meanings of the works that were not obvious at the outset. The positive aesthetic effect of semantic or perceptual ambiguity may be due not just to resolving a conundrum, the authors suggest, but from the “dynamic gain” of multiple insights acquired through the struggle to reach resolution.

The perceived tension between how an object is represented and how we expect it to appear may, in part, be accounted for by the predictive coding model of visual perception (Friston and Kiebel, 2009). Rather than continually adapting to incoming stimuli, this model suggests the visual brain uses prior experience to predict the most likely state of any given scene in advance, and only updating this when a prediction error occurs, that is, when the incoming information contradicts the expected state. Van de Cruys and Wagemans (2011) have usefully applied this paradigm to aesthetic response. They detail a number of cases where artists have more or less deliberately induced prediction errors by the way they have structured their works, leading the viewer to struggle with interpreting the image. They argue this has a generally beneficial effect as far as aesthetic experience is concerned in that expending a certain amount of effort can be pleasurable, especially if this effort is rewarded by solving a perceptual conundrum. In the case of many artworks the “error” between how we would expect an object to appear and how it is represented might trigger a certain state of arousal and so contribute to the aesthetic impact. Another approach is to consider the way artists might subvert the brain's object recognition processing in order to heighten aesthetic impact. In an analysis of the “visual shock” elicited by the disfigured faces and bodies in Francis Bacon's paintings, Zeki and Ishizu (2013) argue the artist violated the brain's templates for face and body recognition. These templates are particularly robust, and therefore more sensitive to distortions than areas of the brain responsible for recognizing human-made artifacts, such as planes and cars. They are also less prone to long-term adaptation, which the authors suggest is why Bacon's deformations can remain disturbing, even after long-term exposure.

On the question of the relationship between art expertise and preference for manifest dichotomy, it is recognized that experts will cognitively process artworks in significantly different ways from non-experts (Cupchik, 1992; Leder et al., 2004). More specifically, recent research found art experts were less emotionally affected by images with a negative or unpleasant valence (Leder et al., 2014). The authors suggest this may be because the aesthetic stance of the art expert is more detached and distanced than the non-expert. Following this line of thought, it may be more likely that art experts can tolerate, or even be aroused by, images or objects that appear difficult, distressing, or confounding to others.

## Implications for the scientific study of aesthetic experience

The hypothesis proposed here is founded on theoretical work carried out in several different disciplines and is consistent with some empirical research in the psychology of aesthetics. I have argued, therefore, the dichotomous nature of artworks should be taken into account in scientific studies of aesthetic experience, certainly where representational artworks are concerned. For those studying the cognitive neuroscience of aesthetic experience there are a number of implications. For one thing, there are obvious questions about the nature of the neurobiological processes associated with the dichotomous experiences described here. How is it, for example, that the mind can be aware of one object having two meanings that are not just distinct but entirely contradictory? Does the neural activity supporting awareness of an object with dichotomous properties differ from that of an object without, and if so how? Do we experience the distinct and incompatible dichotomous states simultaneously, alternately, or discretely? And if dichotomous experiences are inherently irrational in that they are by nature impossible, contradictory or paradoxical, as several researchers claim, then how could we account for this within a rationalist scientific framework?

Mausfeld (2003) and others have suggested the human capacity for appreciating the dichotomous nature of pictures exemplifies a more general feature of human cognition. If this is so, then the fact that it is expressed so markedly in the case of art and aesthetic experience makes it an ideal vehicle for studying this phenomenon in operation. Consider the many other situations where we appreciate dual or contradictory meanings simultaneously. Acting and pretend play have already been mentioned, but also relevant are the many forms of humor that rely on the apprehension of conflicting meaning,^24^ of stage mimics and impressionists who convincingly evoke a sound or personality we know not to be present, of ventriloquist dummies the source of whose voices we simultaneously believe in and disbelieve, of conjurers who perform acts that are apparently possible but also impossible. In all these cases it is necessary we are cognisant of the discrepancy between one reality and another, and it is often the greater discrepancy that elicits the more potent experience.

The phenomenon of the “uncanny valley” is interesting in this regard. Coined by robotics engineer Mori (1970), it refers to the way we can experience negative feelings of unease or eeriness in the presence of a humanoid object that is almost indistinguishable from a real human. Moore (2012) has suggested the effect results from the processing of conflicting cues that on the one hand strongly suggest human-ness and on the other artificiality. His analysis shows that the more uncertain the distinction the greater the sensation of eeriness. This uncanny effect is a form of aesthetic response, which reminds us that aesthetic experience should not be automatically thought of as emotionally positive, or necessarily equated with appreciation of beauty. It is certainly the case with many works that the effect on the viewer is intentionally disorientating, confusing, shocking or repulsive (Leder et al., 2004; Silvia, 2009; Pepperell, 2011; Van de Cruys and Wagemans, 2011).^25^

Much of the preceding literature, therefore, leads to the view that dichotomous, ambiguous, or uncanny stimuli induce special states of mind associated with heightened aesthetic effect. Why should this be so? One suggestion arising from what has been discussed here is that great artworks present us with paradoxical or contradictory information that demands greater attention and requires greater effort to process than do lesser works, so inculcating a stronger sense of involvement with the work on the part of the viewer. It may also be that awareness of the inherent dichotomies embedded in certain artworks is emotionally arousing due to the co-presence of conflicting sensations. This involvement or arousal may not be straightforwardly pleasurable. Indeed, it may be partly unpleasant. But it would create a more intimate bond between viewer and work, and presumably leave a greater impression on the mind, which might be measureable in distinct patterns of neurobiological activity, behavioral response, or memory effects.

A final point to note is that if the dichotomous nature of pictures is an important part of the way we perceive them, as the evidence cited here suggests, then it cannot be overlooked when studying our response to pictures in experimental settings. Neuroscientific studies of face perception, for example, will usually be based on photographic stimuli, which for certain experimental setups is the only practical option. We should therefore be cautious about any conclusions drawn from these studies that do not take the dichotomous pictorial factor into account. For if the aim is to detect the responses to stimuli with great sensitivity, and if the cognitive system is reacting to a dichotomous stimulus in a way it would not be in the presence of a real face, then this may have some bearing on the resulting patterns of neural activation.

## Conclusion

Study of the dichotomous nature of artworks may help us in tackling some long-standing questions about the nature of art and aesthetic experience, such as why we find great works exciting or arousing and what distinguishes them from everyday images or objects. I have argued that with representations of little aesthetic interest, such as undistinguished photographs, the dichotomy between material and meaning passes unnoticed. Most likely this is because we are habituated to the dichotomous effect through long and frequent exposure to representational objects, having learned to cope with their dual nature in early childhood. But as we saw with the examples shown here, certain artworks alert us to the dichotomy in a way that can be crucial to their aesthetic impact. Consequently, the tension or conflict between material support and manifest meaning becomes a factor in the appreciation of the work. Further disparities between the form in which something is represented and our expectations about its appearance will contribute to this aesthetic impact, along with the overall level of complexity induced by the presence of multiple conflicting associations. Artworks that are less aesthetically compelling lack such a diversity of associations.

I have hypothesized that the degree of perceived dichotomy is a factor in the strength of aesthetic response. The greater effort and attention required to process contradictory or paradoxical information will heighted arousal and awareness and so strengthen the bond between viewer and work. In principle, this should be experimentally measurable and the results could provide insights into the neurobiology aesthetic experience.

Many researchers in the sciences and humanities have described the dichotomous properties of pictures and representational objects, and agree we are aware these properties simultaneously, despite the fact they are mutually incompatible. The claim that aesthetic experience is marked by contradictory, paradoxical or impossible states of mind is an extraordinary one since it implies a certain degree of irrationality is involved in such experiences. While artists might be comfortable with this, given they are demonstrably in favor of instilling their works with paradoxical meanings, it may be harder to square with the rationalist scientific framework underpinning neuroaesthetics in particular, and cognitive neuroscience in general.

In order to leave the last word to an artist I close with a brief extract from a conversation between the painter Francis Bacon and the art critic David Sylvester:

Bacon: I want a very ordered image, but I want it to have come about by chance.

Sylvester: It's a matter of reconciling opposites I suppose, of making the thing be contradictory things at once.

Bacon: Well, isn't it that one wants a thing to be as factual as possible and at the same time as deeply suggestive, or deeply unlocking of areas of sensation…? Isn't that what all art is about?^26^

### Conflict of interest statement

The author declares that the research was conducted in the absence of any commercial or financial relationships that could be construed as a potential conflict of interest.

## Footnotes

^1^I am not excluding the possibility that some people experience these two states alternately or sequentially, but have so far found no reports in the literature that they do. Also, on a more general note, the accounts of my experiences when looking at the artworks discussed in this paper are based on seeing them in person. Many of the properties that contribute to aesthetic effects described here are less evident in reproduction.

^2^This example differs from others given here in that the raw materials used, the metal and leather, serve a double function in constituting both bicycle parts and bull-like parts. In the Rembrandt, by contrast, the ink only serves to evoke the artist. This is an important distinction, but not critical to the general argument.

^3^Quoted in Brassai (2002, p. 61).

^4^Paradoxically, we see neither an actual puddle nor just digital marks.

^5^With considerable mental effort I am able to suppress almost entirely the image of the animal-like form and see the bicycle parts for what they are. But I do not seem to be able to do the opposite. It is also interesting to note that when *Bull's* Head was first shown publicly in Paris in 1942 it provoked controversy and was taken off display, suggesting it had a disruptive effect on its audience (Utley, 2000).

^6^I am also able to interpret the Necker cube as a layout of contiguous flat rhomboids, but find it impossible to see it only as a series of black lines.

^7^The Oxford English Dictionary defines “dichotomy” as “division into two sharply defined or contrasting parts; (Logic) division into two mutually exclusive categories or genera.” Note that here that as with all the works discussed we are looking at a reproduction of the painting. This adds at least two further layers of representation, the photograph taken of the work and the medium in which that is reproduced, i.e., digital or mechanical print.

^8^The distinction between “signifier” and “signified” was drawn by the linguist Ferdinand de Saussure, and referred to the form on the one hand and the meaning on the other of the linguistic sign (De Saussure, 2011).

^9^There are many works of art that, arguably, do not present us with the same dichotomy because they are not representational in the commonly understood sense. For example, Marcel Duchamp's *Fountain* (1917) does not purport to stand in for anything other than itself (although this has not prevented critics from comparing its appearance to a seated Buddha). Many entirely abstract paintings lack depictive content, as do many sculptures, performances, happenings, and social interventions. And even certain representational objects reveal no tension between material and referent, as with certain trompe l'oeil paintings, where the material surface is effectively invisible. On this, see Pirenne (1970) and Polanyi (1970).

^10^The word “picture” is used here in the sense of depiction. Images may take many forms, not all of which are depictive.

^11^Gregory, 1970, p. 32.

^12^Gombrich, 1960, p. 236.

^13^Wollheim, 1980, p. 216.

^14^Despite the polarity between Gombrich and Wollheim, a careful reading of the key texts suggests they may have been less far apart than appears. This is because it seems Wollheim misunderstood, or misrepresented, the central claim Gombrich made. Returning to the men-in-a-tunnel example, Gombrich only states something which is clearly true—that we cannot attend to the ink marks as an abstract pattern of lines at the same time as seeing the men in the tunnel. Wollheim takes this to mean we cannot see both the men in the tunnel and the ink marks from which they are made at the same time. But this is not Gombrich's point. Refer to the quotation from *ArtandIllusion* above. What Gombrich means by “see” in this context is not that we do not register the plane surface in our awareness, but that we do not see it *just* as a plane surface—an abstract agglomeration of marks—when we see it as a battle horse. This seems to be quite right, and does not rule out the kind of twofold experience that Wollheim describes. It is a point made lucidly by Bantinaki (2007).

^15^Bell, 1999, p. 26.

^16^Church, 2000, p. 99.

^17^Church would perhaps benefit here from consideration of Wollheim's distinction between seeing-as and seeing-in, where the former has no requirement that we keep the distinction between the represented object and the material support in mind at the same time.

^18^Oxford English Dictionary, ibid.

^19^For an account of the history of this tradition see Stoichita (1997).

^20^As noted, seeing the painting in reproduction adds two further layers: that of the photograph taken from the work and the final means of reproduction, printed or electronic.

^21^The way this work might have been received when first shown is now hard to appreciate. We are used to everyday objects and popular forms such as illustrations and comics being presented as high art. But showing an object in a fine art painting that is depicted in a style more suggestive of commercial illustration would have seemed, at best, incongruous to many people outside avant-garde circles in the early twentieth century, and hence points to a further dichotomy raised in this painting between expectation and delivery.

^22^There may come a point when the disparity between what is represented and the way it is represented becomes so great that recognition fails. For many people unused to interpreting them, the everyday objects in cubist still life paintings can suffer this fate (Muth et al., 2013).

^23^Note that this formulation does not rule out the possibility that non-art objects (such as found objects, kitsch objects, cultural artifacts, designed products, etc.) can have powerful aesthetic impact, providing they still induce multiple contradictory states. See Pepperell (2003) for an argument about the distinction between art works and aesthetically stimulating objects.

^24^According to Weems (2014), the psychologist Richard Wiseman determined the world's funniest joke is one based on a Spike Milligan sketch: “Two hunters are out in the woods when one of them collapses.” He doesn't seem to be breathing and his eyes are glazed. The other guy whips out his phone and calls the emergency services. He gasps, “My friend is dead! What can I do?” The operator says “Calm down. I can help. First, let's make sure he's dead.” There is a silence, then a gun shot is heard. Back on the phone, the guy says “OK, now what?” (p. 26). The contradiction here occurs, of course, between the two meanings of the phrase “make sure he's dead.”

^25^I had a memorable experience of this uncanny effect in the presence of a sculpture by the British artist Gavin Turk, which I viewed at close quarters in a London show. The piece, *Bum* (1998), is a waxwork model of the artist dressed as a tramp, and was so lifelike that it was almost impossible to tell if it was a model or the artist in person holding a very still pose. This uncanniness was a vital part of the work's aesthetic impact.

^26^Francis Bacon in conversation with David Sylvester, ‘Francis Bacon: Fragments of a Portrait’ 18th September 1966, BBC programmes.
